# A Combined Gas and Liquid Chromatographic Approach for Quality Evaluation of Saffron-Based Food Supplements

**DOI:** 10.3390/foods12224071

**Published:** 2023-11-09

**Authors:** Adal Mena-García, María L. Sanz, Marina Díez-Municio, Ana I. Ruiz-Matute

**Affiliations:** 1 Instituto de Química Orgánica General (CSIC), Juan de la Cierva 3, 28006 Madrid, Spain; a.mena@iqog.csic.es (A.M.-G.); ana.ruiz@csic.es (A.I.R.-M.); 2 Pharmactive Biotech Products, S.L.U. Faraday 7, 28049 Madrid, Spain; mdiez@pharmactive.eu

**Keywords:** saffron food supplements, HPLC-MS, GC-MS, crocins, safranal

## Abstract

Considering the interest in the bioactive properties of saffron (*Crocus sativus* L.), as well as its limited production and high price, saffron-based food supplements (SFS) are highly susceptible to adulteration. However, their complex composition and the wide variety of potential fraudulent practices make the comprehensive assessment of SFS quality a challenging task that has been scarcely addressed. To that aim, a new multianalytical strategy based on gas chromatography coupled to mass spectrometry (GC-MS) and liquid chromatography with diode array detection coupled to mass spectrometry (HPLC-DAD-MS) was developed and validated in order to detect different frauds affecting SFS. Dried saffron stigmas and a commercial standardized saffron extract (affron^®^) were selected as reference samples (RS) to obtain an authenticity profile, which was further used to evaluate the quality of 17 SFS. Up to 17 crocins and crocetins, 5 kaempferol glycosides, picrocrocin (determined for the first time by GC-MS), safranal, furanone and isophorone-related compounds were determined in RS. Safranal and crocins were identified in all SFS except for one sample. However, discrepancies with the content declared were detected in 65% of the cases. Moreover, this multianalytical methodology also allowed identifying undeclared additives and the non-declared addition of vegetable sources other than saffron.

## 1. Introduction

Food supplements based on vegetal sources are highly appreciated by consumers as they are perceived as a natural alternative to treat nutrient deficiencies and to promote good health along with physical activity and a balanced diet [1,2]. Therefore, there is a growing demand for plant food supplements (PFS), being one of the fastest growing sectors of industry today, with a high economic impact of both their production and their trade worldwide [1]. This growing trend has been strengthened during the COVID-19 pandemic, as the intake of PFS associated with immune-boosting response or managing anxiety and depression caused by the prolonged isolation and quarantines has increased significantly [1,3]. However, the scarce regulatory controls for PFS commercialization, as well as the limitations of the current official protocols to guarantee their standardization and quality, have led to different fraudulent practices regarding their composition [4,5,6,7].

Saffron is obtained from the dried stigmas of the *Crocus sativus* L. flower, cultivated in many countries, such as Spain, Iran, India, Italy, Greece, and Morocco [8,9]. Due to its multiple bioactive properties (neuroprotective, antidepressant, anti-anxiety, antitumorigenic, cardioprotective, anti-inflammatory, antioxidant, etc.; [10,11]), in recent years, multiple saffron-based food supplements (SFS) have appeared on the market. They are commercialized in different galenic formats (capsules, tablets, etc.), based on saffron as the only vegetal source or as blends with other plants. However, due to its high price, saffron is one of the most adulterated food products [12]. The most common fraudulent practices include the use of material from other plants resembling saffron, such as gardenia, safflower, or turmeric, using the entire flower or other parts of the *C. sativus* plant, such as styles or stamens, beside or instead of stigmas, or using artificial colorants or dyes [12,13,14,15,16,17]. SFS are also very susceptible to fraudulent practices, and thus, the study of different products available on the market is of great interest for both consumers and regulatory authorities in order to assess their quality and identify possible frauds. However, limited studies can be found in the literature concerning SFS quality and authenticity evaluation [15,16]. For this purpose, sensitive and reproducible methodologies must be used to generate the qualitative and quantitative information required for unequivocal fraud detection to allow the determination of a high number of compounds of different nature, such as those present in SFS and in their potential adulterants. 

The official analytical method used for saffron quality determination is the international ISO3632, based on the UV-Visible (UV-Vis) spectrophotometric analysis of its main bioactives, safranal, crocins, and picrocrocin, which are also correlated with the characteristic aroma, color, and flavor of this spice [18,19]. This method is also commonly adapted for the quantitation of saffron bioactives in the standardization process to produce SFS [15]. However, its low selectivity leads to safranal overestimations due to the interferences generated for *cis*-crocins and other compounds also absorbing at the same wavelength [19]. Thus, alternative methodologies based on chromatographic, spectroscopic, molecular-biological, and biomimetic-based techniques have been developed for saffron quality evaluation [20]. Among them, those based on the use of chromatographic techniques (both liquid (HPLC) and gas chromatography (GC)) and their couplings to mass spectrometry (MS), show advantages for their high sensitivity and their separation and identification power [15,18,21,22,23,24]. In this sense, the combined use of chromatographic techniques has been found to be a useful strategy for the geographical discrimination of saffron samples of two different origins (China and Iran) [25]. However, these strategies have not yet been used to assess the quality of more complex samples, such as SFS. 

Therefore, the aim of this study was to develop, for the first time, a multianalytical strategy based on the use of GC and HPLC coupled to MS to establish an authenticity profile that could be used to detect potential fraud in SFS.

## 2. Materials and Methods

### 2.1. Reagents and Standards

Analytical standards of fructose, glucose, gentiobiose, kaempferol-3-*O*-glucoside, maltose, *myo*-inositol, safranal, sucrose, raffinose, verbascose as well as *β*-cyclocitral and phenyl-*β*-glucoside (both used as internal standards, I.S.) were purchased to Sigma Aldrich (St. Louis, MO, USA). Picrocrocin, crocetin bis(gentiobiosyl) ester (crocin *trans*-4-GG) and crocetin gentiobiosylglucosyl ester (crocin *trans*-3-Gg) were provided by Phytolab (Vestenbergsgreuth, Germany). Reagents, such as anhydrous pyridine, trifluoroacetic acid (TFA), hydroxylamine chloride, and hexamethyldisilazane (HMDS), used for derivatization were also acquired from Sigma Aldrich. 

HPLC-MS grade solvents were purchased from Scharlau (Barcelona, Spain). Formic acid was obtained from Sigma Aldrich. Ultrapure water was distilled and purified using Milli-Q, Advantage A10 system from Millipore (Bedford, MA, USA).

### 2.2. Samples

#### 2.2.1. Reference Samples

Three samples of dried commercial extracts of saffron (*Crocus sativus* L.) stigmas (SE1–3), branded as affron^®^, from different batches, were provided by Pharmactive Biotech Products SLU (Madrid, Spain), which are obtained through a proprietary extraction and manufacturing process registered as AFF^®^ ON Cool-Tech. These samples were selected as a reference of standardized extracts used for the manufacture of SFS and are based on Lepticrosalides^®^. According to the granted patents (Ref. US2019099464A1, and Ref. ES2573542B1), Lepticrosalides^®^ is the patented totum of bioactive compounds present in affron^®^ with HPLC safranal content up to 0.35% (dry basis) and HPLC total crocins content up to 6.00% (dry basis) [26,27].

Dried stigmas of *C. sativus* L. samples (DS1–5) were also used as reference samples. These samples were pre-tested according to ISO 3632 [28] and European Pharmacopoeia requirements to confirm their quality. 

#### 2.2.2. Food Supplements

Saffron-based food supplements (SFS1–17) were acquired online or in specialized shops, pharmacies, and supermarkets. Table S1 of Supplementary Materials shows the SFS formulation and composition declared on each label. All SFS were analyzed within their expiration date.

### 2.3. Sample Preparation

Six to ten supplement units per sample were randomly selected, crushed in a porcelain mortar (in the case of tablets), homogenized, and sieved on an Advantech No. 35 sieve (Taipei, Taiwan) of a 500 μm mesh size. 

To evaluate SFS authenticity, reference extracts (*C. sativus* stigmas and affron^®^) were used. Dried stigmas were ground to fine particles in an IKA A10 basic mill (IKA-Werke, Staufen im Breisgau, Germany), sieved through a 500 μm mesh, stored in dry environment and protected from light until extraction. The affron^®^ extract was in powder form and was directly stored in similar conditions.

Regarding HPLC analysis, 20 mg of stigma powder was extracted in 5 mL of methanol/water (50:50, *v*/*v*) using an ultrasonic bath (Elma Schmidbauer GmbH, Singen, Germany) at 27 °C for 10 min (37 KHz, 40% power). For SFS and affron^®^, 100 or 50 mg of the sample were re-dissolved in 2 mL of methanol/water (50:50, *v*/*v*) using the same ultrasound conditions. Samples were then filtered through 0.22 μm PTFE filters (Chromlab S.L., Barcelona, Spain) and immediately stored at −4 °C until analyses were carried out. 

For GC analysis of volatiles, 30 mg of stigma powder was extracted in 2 mL of dichloromethane (DCM) with agitation at 25 °C for 2 h. In the case of affron^®^ extract or SFS, 100 or 200 mg of the sample were re-dissolved in 2 mL of DCM at the same conditions. In all cases, 34 µL of internal standard DCM solution (0.9 mg mL^−1^ of *β*-cyclocitral) was added before extraction.

Regarding GC analysis of low molecular weight polar compounds, reference extracts were obtained by adding 12 mg of stigma powder or 30 mg of affron^®^ to 2.5 mL of water and extracted with agitation at 25 °C for 2 h. On the other hand, 50–100 mg of SFS were also re-dissolved in 2.5 mL of water. Previous to their GC analysis, a derivatization step was required. In brief, 0.05 mL of phenyl-*β*-D-glucoside (1 mg mL^−1^) used as I.S. were added to an Eppendorf with 0.4 mL of reference/SFS extracts and further dried in a rotatory evaporator at 30 °C. The two-step derivatization (oximation + silylation) procedure was carried out as previously described by Zuluaga et al. [29]. For oxime formation, dried samples were treated with 350 μL of 2.5% hydroxylamine chloride in pyridine at 75 °C for 30 min. Silylation was then carried out using 350 μL of HMDS and 35 μL of TFA by heating at 45 °C for 30 min. All derivatized samples were centrifuged at 4401× *g* for 10 min, and the supernatants were further injected into the GC-MS system.

### 2.4. HPLC-MS Analysis

An Agilent 1260 Infinity II Prime LC System with a diode array detector (DAD) and coupled to a 6125 single quadrupole MS detector provided with an electrospray ionization (ESI) source (Agilent Technologies, Santa Clara, CA, USA) was used for analysis of reference samples and SFS. Chromatographic separation was achieved by using a Kinetex PS C18 column (100 × 2.1 mm, 2.6 μm; Phenomenex, Cheshire, UK) thermostatized at 25 °C following a method previously developed [30] and using water (eluent A) and acetonitrile (eluent B), both with 0.01% formic acid, as mobile phase. A complete UV spectrum (210–700 nm) was determined for each peak using DAD detector, and SCAN mode (50–1000 *m/z* range) at positive polarity was followed for MS analyses. Data acquisition and processing were performed using OpenLAB CDS Software (v.2.19.20, Agilent Technologies). 

Compound identification of reference extracts and SFS was based on chromatographic retention and MS data, which was confirmed, when possible, by co-injection of the corresponding commercial standards. A comparison of experimental fragmentation patterns and data provided by the Metlin database (Metabolite and Chemical Entity Database, The Scripps Research Institute, San Diego, CA, USA) or those reported in the literature was also carried out. Identifications were considered tentative when analytical standards were not available.

For quantitative analysis, UV absorbance was measured at 250 nm (picrocrocin and its derivatives), 310 nm (safranal), 350 nm (flavonoids), and 440 nm (crocetin and ester derivatives, crocins). External standard calibration curves of picrocrocin (1–200 µg mL^−1^), safranal (0.4–11 µg mL^−1^), *trans*-4-GG for crocins (1–100 µg mL^−1^), and kaempferol-3-*O*-glucoside for kaempferol glycosides (0.45–90 µg mL^−1^) were used. Precision of the method was measured on the basis of repeatability and intermediate precision. Repeatability and intermediate precision were measured by analyzing affron^®^ reference sample at a concentration of 5 mg mL^−1^ under optimal conditions within the same day (*n* = 5) or in 5 consecutive days for three concentrations. Linearity of the responses was evaluated in the range selected for the calibration curves (Table 1). Goodness of fitting of calibration curves was evaluated using their correlation coefficients. Recovery was calculated in triplicate after spiking at three different concentrations, affron^®^ reference sample, with known amounts of safranal (2, 5, and 9 µg), picrocrocin (5, 16, and 50 µg), and *trans*-4-GG crocin (16, 25, and 50 µg) standards. Limits of detection (*LOD*) and quantitation (*LOQ*) were calculated for safranal, picrocrocin, and *trans*-4-GG crocin as three and ten times the signal-to-noise ratio, respectively.

For further structure confirmation of these identifications and characterization of unknowns, HPLC-MS/MS analyses were carried out in an Agilent 1200 Series LC system (equipped with a binary pump, an autosampler, and a column oven) coupled to a 6520 quadrupole-time of flight (QToF) mass spectrometer (Agilent Technologies). Compound separation was carried out using the Kinetex PS C18 column under gradient conditions described above using a flow rate of 0.1 mL min^−1^. The MS analysis was performed with ESI interface working in positive ion mode polarity, electrospray voltage set at 4.5 kV, fragmentor voltage at 150 V, and the drying gas temperature at 300 °C. Nitrogen (99.5% purity) was used as nebulizer (30 psi) and drying gas (6 L min^−1^), whereas nitrogen of higher purity (99.999%) was used as collision gas. Tandem mass spectra were obtained by collision-induced dissociation (CID), applying collision energies between 10 and 30 eV to the selected precursor ions. Data acquisition and processing were performed using Agilent Mass Hunter Workstation Rev. B.02.00 software. Mass accuracy below 5 ppm was obtained for elemental composition of target compounds.

### 2.5. GC-MS Analysis

A 6890 N gas chromatograph coupled to a 5973 single quadrupole mass spectrometer, both from Agilent Technologies (Palo Alto, CA, USA), were used for the analysis of derivatized (aqueous extracts) and non-derivatized samples (DCM extracts). A Zebron ZB-1 capillary column (30 m × 0.25 mm i.d.; 0.25 μm film thickness; Phenomenex, CA, USA), helium as carrier gas at 0.8 mL min^−1^ and an injection volume of 1 μL were used. Different operating conditions (oven programs, injection T, and split ratio) were applied for each type of sample. For derivatized samples, injection T and split ratio were set at 320 °C and 1:20, respectively. Oven T started at 180 °C and ramped at 15 °C min^−1^ to 320 °C (stayed for 1 min) and then ramped at 40 °C min^−1^ to 330 °C (stayed for 30 min). For non-derivatized samples, injection T and split ratio were set at 250 °C and 1:10, respectively. Oven T started at 80 °C and ramped at 15 °C min^−1^ to 140 °C, then ramped at 30 °C min^−1^ to 250 °C (stayed for 1 min) and finally ramped at 50 °C min^−1^ to 300 °C (stayed for 6 min). The MS detector, in both cases, was operated in electron impact (EI) mode at 70 eV, scanning the 50–650 *m/z* range. The transfer line was set at 280 °C and the ionization source at 230 °C. For data acquisition and analysis, MSD ChemStation software E.02.02.1431 (Agilent Technologies) was used. Identifications were carried out by comparing experimental spectra with data from mass spectral libraries (Wiley, NIST). The internal standard method was used for quantitation. Standard solutions of safranal (0.6–50 µg mL^−1^), picrocrocin (7–165 µg mL^−1^), fructose (7–400 µg mL^−1^), glucose (7–365 µg mL^−1^), *myo*-inositol (7–150 µg mL^−1^), sucrose (9–212 µg mL^−1^), maltose (9–920 µg mL^−1^), raffinose (10–500 µg mL^−1^), and verbascose (10–250 µg mL^−1^) were used to calculate the response factor relative to the internal standard (phenyl-*β*-D-glucoside for derivatized compounds and *β*-cyclocitral for underivatized compounds). Quantitation of compounds for which commercial standards were not available was carried out using the response factor of their isomers (safranal for terpenoid volatiles, maltose for gentiobiose, raffinose for maltotriose, and verbascose for maltotetraose). All analyses were carried out in duplicate unless otherwise stated. Safranal and derivatized picrocrocin in affron^®^ samples were considered for the determination of analytical parameters of the methodologies such as precision, linearity, and goodness of the fitting of the calibration curve, recovery, *LOD*, and *LOQ*, as described in Section 2.4. 

### 2.6. Statistical Analysis

Statistical analysis was carried out using the Statistica 7.0 program (StatSoft, Inc., Tulsa, OK, USA). The compliance between the experimental and declared values of safranal and crocins were assessed by *t*-test (*p* < 0.01), and the quality of SFS was evaluated by Principal Component Analysis (PCA). 

## 3. Results

### 3.1. Analysis of Reference Extracts

In order to obtain a multicomponent authenticity profile which could be useful to assess the quality and genuineness of SFS, dried saffron stigma extracts (DS1–5) and standardized commercial saffron extracts (SE1–3) were analyzed by different chromatographic techniques (HPLC-DAD-MS, HPLC-QToF MS, and GC-MS). All these analyses provided qualitative and quantitative data on their composition in volatiles, low molecular weight carbohydrates (LMWC), and apocarotenoids. 

#### 3.1.1. Qualitative Analysis

HPLC analysis allowed the characterization of higher molecular weight polar compounds from reference extracts. The fragmentation pattern obtained by HPLC-MS/MS analysis was particularly useful for the identification of several compounds from different families, such as crocins, flavonoid glycosides, and picrocrocin. Due to the lack of most standards, identifications were carried out based on the relative peak intensities, elution order, and fragmentation patterns reported in the literature [16,18,31,32]. 

**Figure 1:**
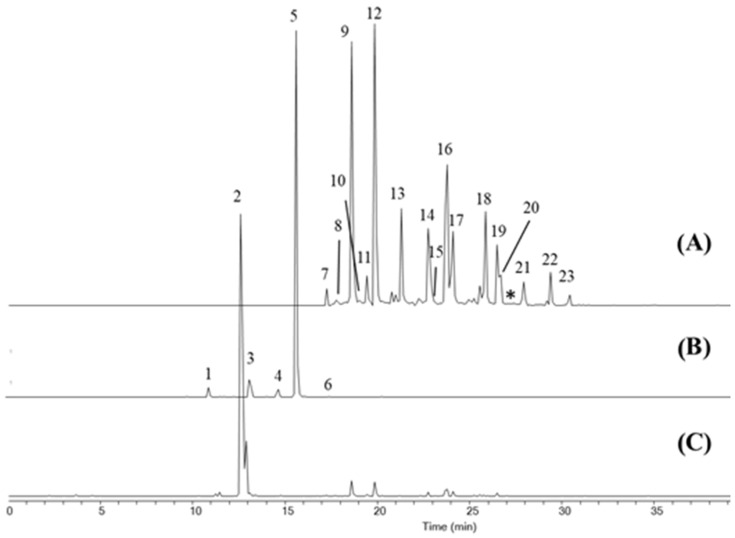
Extracted Ion Cromatograms (EIC) selected for saffron bioactive compounds in sample SE1 acquired by HPLC-QToF MS: (**A**) crocins (*m/z* ion at 329.1747), (**B**) kaempferol derivatives (*m/z* ion at 611.1640), and (**C**) picrocrocin (*m/z* ion at 331.1761). (1) kaempferol-3-sophoroside-7-glucoside, (2) picrocrocin, (3) kaempferol triglucoside, (4) kaempferol-7-sophoroside or kaempferol-3,7-diglucoside, (5) kaempferol-3-sophoroside, (6) kaempferol-3-glucoside, (7) *trans*-5-tG, (8) *trans*-4-GG, (9) *trans*-4-GG, (10) *trans*-4-tg, (11) *trans*-4-ng, (12) *trans*-3-Gg, (13) *trans*-2-gg, (14) *cis*-4-GG, (15) *cis*-4-ng, (16) *trans*-2-G, (17) *cis*-3-Gg, (18) *cis*-2-gg, (19) *trans*-1-g, (20) *cis*-2-G, (21) *cis*-1-g, (22) *trans*-crocetin, and (23) *cis*-crocetin. * safranal elution time.

Regarding flavonoid glycosides, five compounds could be identified as derivatives of kaempferol based on their DAD signal at 350 nm and MS data (Figure 1). Peaks 1 and 3, showed a signal at *m/z* ion 773.2127 compatible with a molecular formula of [C_33_H_40_O_21_ + H]^+^ with good accuracy (exact mass error of 0.06 ppm), corresponding to kaempferol derivatives with three glucose units. They were tentatively identified as kaempferol-3-sophoroside-7-glucoside and kaempferol triglucoside based on that reported by other authors [15,16,32]. A similar fragmentation pattern was observed for both, with *m/z* ions at 611.1612, 449.1087, and 287.0543, corresponding to different loses of glucose (Glc) units: [M + H-Glc]^+^; [M + H-2Glc]^+^ and [M + H-3Glc]^+^. Peak 4 was identified as kaempferol-7-sophoroside or kaempferol-3,7-diglucoside and peak 5 as kaempferol-3-sophoroside, with a common *m/z* 611.1640 ion corresponding to [C_27_H_30_O_16_ + H]^+^ species and fragments at 449.1090 and 287.0567 corresponding to [M + H-Glc]^+^; [M + H-2Glc]^+^ [15,16,29]. Finally, kaempferol-3-glucoside (C_17_H_20_O_11_) was identified as peak 6 (*m/z* ions at 449.1088 [M + H]^+^ and 287.0536 [M + H-Glc]^+^). Picrocrocin was assigned as peak 2 in both types of reference samples by comparing its retention time, DAD, and MS spectra with that of its standard. 

Although different ESI parameters (fragmentor voltage, ESI voltage, nebulizing gas pressure, and drying gas temperature) were assayed in order to optimize safranal ionization, a very low MS signal was obtained. Nevertheless, it could only be correctly identified using its DAD signal at 310 nm and by comparing its retention time with that of its commercial standard, eluting between peaks 20 and 21 (marked with an asterisk in Figure 1).

Finally, as indicated in our previous work [30], up to 17 isomers of crocins (crocetin esters) and crocetin (peaks 7 to 23), corresponding to the *trans*- and *cis*-forms, could be identified in both reference samples, DS and SE, in which a common *m/z* 329.1747 ion corresponding to crocetin [M + H]^+^ species was observed. Moreover, characteristic fragment ions corresponding to different carbohydrate cleavages were also detected, which were used for their identification. Among them, the highest molecular weight crocetin esters identified were those with five glucose units (*trans*-5-tG and *trans*-5-nG, peaks 7 and 8), while the lowest consisted of crocetin structures without any glycosylation (peaks 22 and 23). 

**Figure 2:**
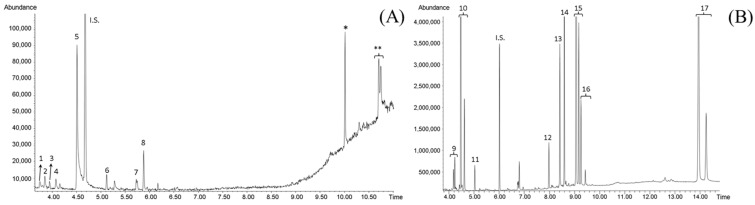
GC-MS chromatograms of affron^®^ reference extract in dichloromethane (**A**) and water after derivatization (**B**). (1) Dihydro-4-hydroxy-2(3H)-furanone, (2) isophorone, (3) ketoisophorone, (4) dihydrooxophorone, (5) safranal, (6) 4-hydroxy-isophorone, (7) 4-hydroxy-2,6,6-trimethyl-3-oxocyclohexa-1,4-diene-1-carboxaldehyde, (8) HTCC, (9) fructose, (10) glucose, (11) *myo*-inositol, (12) sucrose, (13) unknown glycoside; (14) picrocrocin, (15) maltose, (16) gentiobiose, and (17) maltotriose. *: artefact; ** HTCC derivative. I.S.: Internal Standard.

Regarding GC-MS analyses, the volatile composition of reference samples was directly determined by analyzing DCM extracts, while LMWC and some glycosides were detected in the derivatized aqueous extracts. As Figure 2A shows, a similar volatile profile was obtained for SE1 in comparison with saffron stigmas (Figure S1), where characteristic volatiles of saffron could be detected. Safranal (peak 5) was the main peak followed by HTCC (4-hydroxy-2,6,6-trimethylcyclohex-1-enecarbaldehyde, peak 8). Other furanone or isophorone-related compounds were also detected, such as dihydro-4-hydroxy-2(3H)-furanone (peak 1), isophorone (3,5,5-trimethyl-2-cyclohexen-1-one, peak 2), ketoisophorone (2,6,6-trimethyl-2-cyclohexene-1,4-dione, peak 3), dihydrooxophorone (2,6,6-trimethyl-1,4-cyclohexanedione, peak 4), 4-hydroxy-isophorone (4-hydroxy-3,5,5-trimethylcyclohex-2-enone, peak 6), and 4-hydroxy-2,6,6-trimethyl-3-oxocyclohexa-1,4-diene-1-carboxaldehyde (peak 7). Moreover, two compounds with similar mass spectrum (107, 123, 151, and 168 *m/z* ions) to that of HTCC (** in Figure 2A) were also found at longer retention times and were tentatively identified as HTCC derivatives. All these compounds have been reported in the literature by different authors as the major components of saffron aroma [8,33,34,35].

Several LMWC were also detected in the aqueous derivatized reference samples by GC-MS, such as fructose, glucose, *myo*-inositol, and sucrose (peaks 9–12 in Figure 2B). These compounds, identified in both DS and SE samples, are commonly found as natural components in plants [29,36,37]. In particular, *myo*-inositol has been previously described as a genuineness marker of the natural origin for other plant food supplements [6,38]. Also, gentiobiose (peak 16), a disaccharide present as one of the main glycoside moieties in crocin structure [39,40], was identified in both DS and SE. Contrarily, maltose and maltotriose (peaks 15 and 17) were only detected in SE samples, and their presence was associated with the maltodextrins used as stabilizers and bulking agent during their fabrication process. It must be highlighted that peaks 13 and 14 also presented mass spectra compatible with a typical glycoside fragmentation, with fragment ions at *m/z* 204 (typical of pyranose rings) and 361 (related to glycosidic linkages) [41]. The characteristic *m/z* ion at 237 observed in peak 14 was associated with picrocrocin (*β*-D-glucoside of *β*-cyclocitral) structure, which corresponds to the trimethylsilyl oxime of the aglycone after losing the glucose moiety (Figure S2). The confirmation of picrocrocin identification was carried out by comparing its retention time and mass spectrum with that of its pure standard. This compound, a precursor of safranal, is responsible for the bitter taste of saffron and has been described as an authenticity biomarker of saffron [42,43]. It must be highlighted that, to the best of our knowledge, this is the first time that picrocrocin has been determined by GC-MS after its derivatization, which could be an alternative method to determine this marker compound.

#### 3.1.2. Quantitative Analysis

For the quantitation of the main bioactive compounds (safranal, picrocrocin, and crocins) in saffron reference samples, the different analytical methodologies used were previously validated. Both HPLC-DAD and GC-MS were evaluated for the quantitation of safranal and picrocrocin while crocins were determined by HPLC-DAD. 

As shown in Table 1, several analytical parameters were considered. A good linearity was obtained for the relationship between peak areas and concentrations in the ranges evaluated for each compound, with coefficients of determination (R^2^) higher than 0.997 in all cases. Regarding analytical precision, the repeatability obtained for all compounds was good, with RSD values of 3.9% for crocin *trans*-4-GG, 1.6 and 2.9% for picrocrocin, and 3.9 and 6.7% for safranal by HPLC-DAD and GC-MS, respectively. Furthermore, intermediate precision was also calculated, with RSD values lower than 4.7% for trans-4-GG, safranal and picrocrocin by HPLC-DAD and 6.6% and 9% for picrocrocin and safranal by GC-MS. Concerning recoveries, although good values were obtained at the different spiking concentrations for all target compounds, better results were obtained for HPLC-DAD, especially in the case of safranal (99–101% vs. 91–98% by GC-MS). A slightly higher sensitivity was also observed for HPLC-DAD for safranal and picrocrocin based on the results obtained for their limits of detection (*LOD*) and quantitation (*LOQ*) (*LOD*_safranal_: 2.2 vs. 10.5 ng and *LOD*_picrocrocin_: 12.7 vs. 50 ng for HPLC-DAD and GC-MS, respectively). Thus, although the different methodologies proved to be suitable for the determination of all the compounds evaluated, HPLC-DAD showed better analytical performance based on its higher sensitivity and higher recovery results, and it was selected for safranal, picrocrocin, and crocins quantitation of reference samples (Table S2).

As for picrocrocin content, saffron stigmas (DS1–5) showed mean values between 74.7 and 105.5 mg g^−1^ of saffron, while safranal ranged from 2.52 to 3.30 mg g^−1^ of saffron, which are within the same concentration ranges reported by other authors [44,45,46]. The average values for total crocins varied between 138.6 and 167.7 mg g^−1^ of saffron, being *trans*-isomers more abundant than the *cis*-forms. Among them, and according to that reported in the bibliography [18,30,31,47], *trans*-4-GG was the most abundant isomer, followed by *trans*-3-Gg. A wide range of values has been reported for crocins in saffron (e.g., from 29 mg g^−1^ [48] up to 549 mg g^−1^ [46]). These amounts vary greatly depending on multiple factors, such as geographical origin, post-harvest practices, etc. [47,48], being the values determined in DS1–5 within this range. Kaempferol-3-sophoroside was found to be the most abundant flavonoid glycoside (13.0–14.3 mg g^−1^ of saffron) in DS, followed by kaempferol-3-sophoroside-7-glucoside (5.5–6.7 mg g^−1^ of saffron). These concentrations were in accordance with those previously reported for saffron stigmas by Masi et al. [46] (5.0–10.4 mg g^−1^ of saffron for kaempferol-3-sophoroside and 2.6–5.2 mg g^−1^ of saffron for kaempferol-3-sophoroside-7-glucoside) and slightly higher than those reported by Carmona et al. [49] (0.6–3.1 mg g^−1^ of saffron for kaempferol-3-sophoroside and 1.5–2.6 mg g^−1^ of saffron for kaempferol-3-sophoroside-7-glucoside). In addition, other kaempferol glycosides (compounds 3, 4, and 6 in Table S2) were determined. However, only concentration data about kaempferol triglucoside in saffron stigmas were found in the bibliography [46,49], which agrees with that reported in Table S2. 

Lower amounts of all these saffron bioactives were observed for commercial standardized saffron extracts (affron^®^, SE1–3, Table S2). These products, used for the manufacture of food supplements, are obtained after standardization processes of stigma extracts [30] and diluting with other substances (such as maltodextrins). In these samples, safranal varied from 0.27 to 0.5 mg g^−1^ of extract, while picrocrocin, total crocins, and total kaempferol glycosides were in the range of 29.3–32.6, 39.4–42.8, and 6.3–6.9 mg g^−1^ of extract, respectively. 

Data obtained by GC-MS for volatile compounds (other than safranal) or derivatized carbohydrates present in reference samples are included in Table S3. The highest concentrations of volatiles in saffron stigmas were obtained for HTTC (from 310 to 591 µg g^−1^ saffron), 4-hydroxy-2,6,6-trimethyl-3-oxocyclohexa-1,4-diene-1-carboxaldehyde (170 to 260 µg g^−1^ saffron), isophorone (ranging from 202 to 245 µg g^−1^ saffron) and dihydrooxophorone (178–208 µg g^−1^ saffron). The same major saffron volatile compounds, along with safranal, have been described in the literature [8,9,33,34,35]. However, a large variability in their concentrations has been reported, not only to post-harvest or geographical origin factors but also to the procedures associated with their extraction and characterization [9,22,50]. The lower concentrations of volatiles detected in commercial saffron reference extracts (SE1–SE3) (about 20 times lower than those of saffron stigmas, Table S3) could be explained by their loss as a result of standardization and manufacturing processes discussed above.

Among LMWC, glucose, the main glycosidic monomer of crocins, was the most abundant in both DS and SE samples (31–50 and 5.5–12 mg g^−1^ of saffron, respectively), followed by fructose and gentiobiose in DS (12–13 and 3.4–8.2 mg g^−1^ of saffron, respectively). The high values of maltotriose and maltose determined in SE were due to the addition of maltodextrins to these products, as commented before. 

### 3.2. Food Supplement Analysis

Once the qualitative and quantitative profiles of reference samples were evaluated, a comprehensive characterization of different saffron food supplements (SFS1–17) was performed on the basis of data obtained by both chromatographic techniques (GC-MS and HPLC-DAD-MS) in order to assess their quality and authenticity by comparing: (i) with the information given in their label, (ii) SFS chromatographic profiles with that obtained for reference samples, and (iii) quantitative data of all samples in a multivariate analysis (PCA). 

As shown in Table S1, among all the samples analyzed in this study, 88% declared concentrations of saffron bioactive compounds on their label (safranal and/or total crocins). Experimental values obtained for these compounds by HPLC-DAD, expressed as the percentage of declared saffron extract, were compared with those declared on the label (Table 2). Although most SFS declared a safranal content of 2%, experimental concentrations found in these samples were much lower, ranging from traces (SFS3) to 0.157% (SFS15). These discrepancies found between the experimental and labeled safranal content could probably be related to the methodology used for the quantitation of this bioactive during the standardization process of saffron products, where the lack of selectivity of the commonly used ISO3632 methodology (based on UV spectrophotometry determinations) leads to its overestimation [15]. However, a good agreement between experimental and declared values was found for those SFS that declared 0.03% of safranal.

Regarding total crocin concentrations, a high variability (from 0.182% in SFS10 to 8% in SFS9, Table 2) was observed for the different food supplements, which can be explained by the fact that most products were not standardized considering these compounds. This high variability in crocins content was also observed by other authors [15,16], ranging between 0.3% and 15.8%. Among the supplements for which crocin concentrations were reported, significant differences were found for sample SFS10, which had a total crocin concentration ten times lower than the declared value. It should be noted that neither safranal nor crocins were detected in SFS6, raising suspicions about its authenticity.

**Figure 3:**
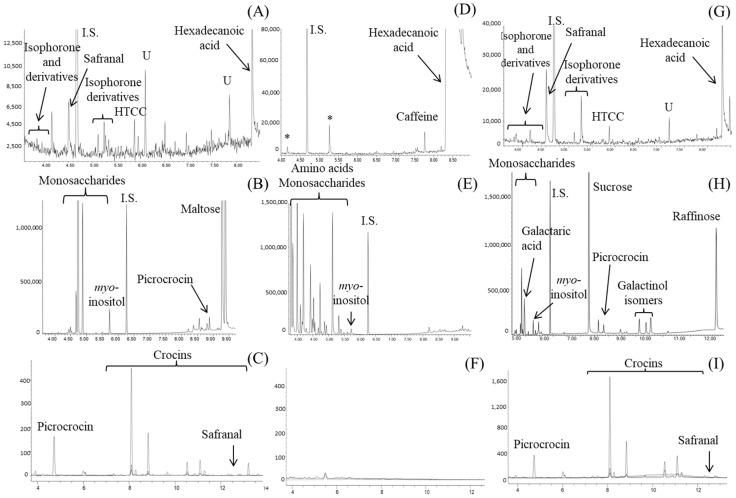
GC-MS profiles obtained for: (i) volatile composition of SFS1 (**A**), SFS6 (**D**) and SFS9 (**G**) and (ii) derivatized carbohydrates of SFS1 (**B**), SFS6 (**E**) and SFS9 (**H**); and HPLC-DAD profile at 250, 310, and 440 nm for SFS1 (**C**), SFS6 (**F**), and SFS9 (**I**). I.S.: Internal Standard. U: unknowns; *: artifacts.

When considering the multianalytical profiles of SFS, in general, they were similar to those obtained for reference samples, with some exceptions. As an example, Figure 3A–C shows the GC-MS and HPLC-DAD-MS profiles for SFS1, labeled to contain only saffron extract and excipients (Table S1). Regarding its volatile composition, a poor profile was obtained, where safranal was the major compound (Figure 3A). Other characteristic saffron compounds, such as HTTC, 4-hydroxy-2,6,6-trimethyl-3-oxocyclohexa-1,4-diene-1-carboxaldehyde, and isophorone derivatives, could also be determined, although in very low amounts (Table 3). This is in line with the volatile loss observed for the standardized reference extracts (SE) in comparison to dried stigmas (DS) but at a higher extent due to dilution steps with excipients and the additional processing stages involved.

When this sample was subjected to derivatization and analyzed by GC-MS (Figure 3B, Table 4), carbohydrates and glycosides previously detected in reference samples were also found, highlighting the presence of *myo*-inositol and picrocrocin as the most important markers. It must be noted the high concentrations found for maltodextrin-related carbohydrates, such as maltose, maltotriose, and maltotetraose (Table 4), compared to those observed in the reference SE. As for the HPLC-DAD-MS profile (Figure 3C, Table 5), the same bioactive compounds as those detected in reference extracts were found, although in lower concentrations. 

Sample SFS3, containing up to seven plant extracts other than saffron and five other ingredients, such as carotenoids or coenzyme Q10, was particularly a complex case (Table S1). This resulted in very complicated chromatographic profiles, especially for HPLC-DAD-MS, where different crocins could be determined, but only traces of safranal and even the absence of picrocrocin were observed. However, these compounds could be detected by GC-MS, confirming the presence of saffron extract in this SFS. This proves the utility of the developed multianalytical strategy for the determination of saffron food supplement authenticity. 

In the case of supplement SFS 4, which was also composed of a mixture of different plant extracts, bioactive compounds of saffron were correctly identified and quantified by the developed methods (Table 3, Table 4 and Table 5), such as isophorone and its derivates (between 4.6 and 12.2 µg g^−1^ of SFS), HTCC (34 µg g^−1^ of SFS), picrocrocin (3.4 mg g^−1^ of SFS), and crocins (from traces to 2.9 mg g^−1^ of SFS). Moreover, bioactive compounds characteristic of the other declared plant sources were also identified by the developed methodologies, such as hydroxycitric acid for *Garcinia cambogia* [38,51] and caffeine and chlorogenic acids for green coffee [52]. 

As already mentioned, safranal and crocins were not detected in SFS6, but also other characteristic compounds of saffron were not found (Table 2, Table 3 and Table 5, Figure 3D–F). Only *myo*-inositol and some sugars were detected, which could indicate the use of a plant extract other than saffron in the formulation of this sample. Moreover, although several vitamins and two amino acids were declared in its label (Table S1), only tryptophan and vitamin B6 were identified after their derivatization by GC-MS (Figure 3E). 

It should be noted that various undeclared compounds could also be detected in some of the SFS analyzed. As an example, chromatograms of SFS8 are shown in Figure 3G–I, where volatile composition (Figure 3G) and chromatographic profile by HPLC-DAD-MS (Figure 3I) were similar to that of reference samples, but the profile obtained by GC-MS for the derivatized sample (Figure 3H) showed several unexpected compounds. The presence of galactinol, galactinol isomers, and raffinose, which have been described as common compounds in leguminous [53,54], combined with a high abundance of sucrose, could indicate the undeclared addition of a legume-derived ingredient in SFS8 sample. 

Maltose, maltotriose and/or maltotetraose were detected in 16 of the samples analyzed in a wide concentration variability (Table 4), which could indicate the probable presence of maltodextrins, used as excipients; however, only half of them declared these compounds on the label. 

Furthermore, caffeine was detected in samples SFS6 and SFS11, but it was not declared in their labels. While a high abundance of undeclared caffeine could indicate the purposeful addition of this synthetic compound for a desired pharmacological activity [55], the low abundances found in these supplements could be due to cross-contamination from other products or natural sources. In addition, a contaminant formed during heating or preservation processes, known as 5-hydroxymethylfurfural (HMF), could be found at trace levels in SFS6.

Lastly, in order to explore the potential benefits of a multi-analytical strategy to assess the quality of SFS, PCA was applied to a matrix of combined HPLC-DAD and GC-MS data for SFS and reference samples (SD and SE). Strong correlations were observed between compounds of the same family when used individually as variables (data not shown), so new variables were created based on the sum of structurally related compounds (kaempferol glycosides, crocins, volatiles, monosaccharides, disaccharides, and sugars from maltodextrins), while other individual compounds remained as unchanged variables (*myo*-inositol, picrocrocin, and safranal). Moreover, sample SFS6, which did not show any of the bioactives characteristic of saffron was not included in this study. 

As can be seen in PC1 vs. PC2 plot (Figure S3), the first two principal components explained more than 92% of the total variance, being the content of *myo*-inositol and the sum of kaempferol glycosides the variables with more weight in sample segregation on PC1 with negative scores (−0.9951 and −0.9939, respectively), and maltodextrins and disaccharides on PC2 with a negative score (−0.7549) and a positive score (0.6732), respectively. While reference samples were grouped in independent clusters (I and II, respectively), a different behavior was observed depending on the food supplement considered. In the case of SF9, it was positioned close to reference samples (cluster II) due to its higher concentrations of characteristic saffron compounds when compared to the other commercial extracts, which could indicate the possible addition of ground saffron stigma to these sample. The high weight of carbohydrate content in sample segregation was observed in the case of SFS3 and SFS8 (group IV), supplements with a high content of sucrose (8.2 and 6.9 mg g^−1^, respectively) and in samples grouped in cluster V, with high concentrations of maltodextrins (from 146.5 to 435.9 mg g^−1^). Other SFS were included in group III. 

## 4. Conclusions

The great potential of the combined use of chromatographic techniques (GC-MS and HPLC-DAD-MS) and chemometric tools for the evaluation of the quality and authenticity of saffron food supplements has been demonstrated. In addition, to the best of our knowledge, this is the first time that picrocrocin has been reported by GC-MS and that comprehensive profiles (volatiles, LMWC, and other polar bioactive compounds) of saffron food supplements have been reported. The comparison of saffron authenticity profiles with those obtained for the SFS allowed the detection of different types of frauds, such as additions of undeclared ingredients or other natural sources. Moreover, discrepancies between the concentration of bioactive compounds declared on the label and those experimentally determined were also found. These results highlight the need for sensitive and selective methodologies, such as those proposed in this work, to control and ensure the quality of the products available on the market.

## Figures and Tables

**Table 1:** Validation parameters for HPLC-DAD and GC-MS methods for the main saffron bioactives.

	Safranal		Picrocrocin		*trans*-4-GG
	HPLC-DAD	GC-MS	HPLC-DAD	GC-MS ^#^	HPLC-DAD
Calibration curve	y = 34.98x − 0.36	y = 1.26x − 0.03	y = 14.29x + 4.69	y = 0.55x − 0.04	y = 58.75x + 7.22
	R^2^ = 0.9994	R^2^ = 0.9991	R^2^ = 0.9993	R^2^ = 0.9965	R^2^ = 0.9997
Linear range (µg mL^−1^)	0.4–11	0.6–50	1–200	7–165	1–100
Repeatability (% RSD)	3.9	6.7	1.6	2.9	3.2
Intermediate precision (% RSD)	4.7	9.0	2.2	6.6	3.6
*LOD* (ng)	2.18 ± 0.04	10.5 ± 0.1	12.7 ± 0.6	50 ± 10	2.04 ± 0.02
*LOQ* (ng)	7.3 ± 0.1	35 ± 0.4	42 ± 2	200 ± 32	6.79 ± 0.05
Recovery (%)					
Addition 1 *	100 ± 3	91 ± 3	101.1 ± 0.6	97 ± 4	101.8 ± 0.6
Addition 2	101 ± 3	97.8 ± 0.9	101 ± 2	107 ± 9	102 ± 2
Addition 3	98.9 ± 0.6	95.3 ± 0.9	98 ± 2	94 ± 5	100 ± 2

* Addition 1, 2 and 3: safranal (2, 5, and 9 μg); picrocrocin (5, 16, and 50 μg) and *trans*-4-GG (16, 25, and 50 μg). ^#^ Derivatized picrocrocin.

**Table 2:** Comparison of the percentage of safranal and crocins declared in SFS labels and the experimental value determined by HPLC-DAD. Standard deviations in parentheses.

	Safranal (%, *w*/*w*)		Crocins (%, *w*/*w*)	
	Experimental	Declared	Experimental	Declared
SFS1	0.049 (0.004)	0.03	2.90 (0.06)	3.48
SFS2	0.020 (0.002)	0.03	1.8 (0.1)	3.48
SFS3	<*LOQ*	N.D ^$^	0.72 (0.02)	N.D
SFS4	0.0972 * (0.0008)	2	5.2 (0.2)	N.D
SFS5	0.0231 * (0.0006)	2	6.0 (0.1)	N.D
SFS6	- ^#^	N.D	-	N.D
SFS7	0.016 * (0.002)	2	3.3 (0.4)	N.D
SFS8	0.023 (0.001)	0.03	2.00 (0.06)	3.48
SFS9	0.109 * (0.004)	2	8.0 (0.3)	N.D
SFS10	0.001710 *(0.000004)	2	0.182 * (0.004)	2
SFS11	0.092 * (0.005)	2	1.7 (0.1)	N.D
SFS12	0.082 * (0.003)	2	1.24 (0.01)	N.D
SFS13	0.143 * (0.002)	N.D	3.45 (0.01)	3
SFS14	0.049 * (0.001)	2	2.50 (0.03)	3
SFS15	0.157 * (0.007)	2	2.9 (0.1)	N.D
SFS16	0.105 * (0.003)	2	3.29 (0.03)	4
SFS17	0.0435 (0.0007)	0.03	2.14 (0.04)	3.48

^#^ non-detected. ^$^ non-declared. * Means statistically different from the theoretical value (declared) at a *p* < 0.01 probability level.

**Table 3:** Volatile compounds quantified by GC-MS (µg·g^−1^) in SFS1–17. Standard deviations in parentheses.

									SFS								
	1	2	3	4	5	6	7	8	9	10	11	12	13	14	15	16	17
Dihydro-4-hydroxy-2(3H)-furanone	- ^#^	-	-	-	-	-	0.9 (0.08)	-	15 (8)	0.24 (0.03)	-	-	-	-	-	18 (2)	-
Isophorone	0.56 (0.07)	0.98 (0.08)	-	10.4 (0.1)	-	-	0.56 (0.02)	2.6 (0.5)	70 (20)	1.5 (0.2)	1.4 (0.3)	-	2.6 (0.5)	7.3 (0.2)	-	18 (1)	1.8 (0.09)
Ketoisophorone	0.37 (0.02)	0.62 (0.02)	3.9 (0.3)	4.6 (0.6)	-	-	0.58 (0.03)	-	53 (6)	4.7 (0.4)	1.1 (0.3)	-	2.9 (0.4)	5.0 (0.5)	-	21.9 (0.02)	0.89 (0.04)
Dihydrooxophorone	0.52 (0.04)	0.772 (0.003)	-	8.79 (0.02)	-	-	1.2 (0.2)	1.2 (0.5)	60 (10)	3.0 (0.3)	-	-	2.6 (0.2)	6.0 (0.2)	-	14.7 (0.5)	1.55 (0.03)
4-Hydroxy-isophorone	0.78 (0.04)	0.55 (0.05)	-	12.2 (1.0)	1.6 (0.2)	-	2.1 (0.2)	2.4 (0.6)	50 (20)	7.0 (0.5)	1.5 (0.2)	-	2.5 (0.1)	9.7 (0.3)	-	19.50 (0.02)	1.52 (0.01)
4-Hydroxy-2,6,6-trimethyl-3-oxocyclohexa-1,4-diene-1-carboxaldehyde	2.3 (0.5)	2.267 (0.006)	-	15 (2)	2.6 (0.6)	-	5 (1)	-	80 (40)	1.4 (0.4)	-	-	-	9 (2)	-	11.2 (0.5)	1.85 (0.04)
HTCC	3.0 (0.4)	2.5 (0.2)	11.7 (0.2)	34 (1)	4.13 (0.05)	-	7.7 (0.9)	4.1 (0.8)	150 (80)	1 (1)	-	-	-	14 (1)	-	39 (3)	3.39 (0.06)

^#^ Non detected.

**Table 4:** Low molecular weight carbohydrates quantified by GC-MS (mg·g^−1^) in SFS1–17. Standard deviations in parentheses.

							SFS										
	1	2	3	4	5	6	7	8	9	10	11	12	13	14	15	16	17
Fructose	0.16 (0.01)	0.183 (0.008)	1.9 (0.2)	0.394 (0.001)	0.51 (0.01)	1.8 (0.3)	0.31 (0.05)	0.5 (0.1)	4.2 (0.3)	0.230 (0.004)	0.29 (0.04)	0.153 (0.02)	- ^#^	0.092 (0.002)	0.21 (0.03)	1.01 (0.05)	1.44 (0.04)
Glucose	5.3 (0.3)	5.22 (0.05)	2.1 (0.2)	2.02 (0.05)	9.2 (0.1)	2.7 (0.3)	0.5 (0.1)	2.3 (0.2)	11.6 (0.6)	3.26 (0.04)	2.4 (0. 2)	2.3 (0.1)	-	0.46 (0.06)	3.0 (0.2)	2.5 (0.2)	2.5 (0.2)
*myo*-Inositol	0.032 (0.004)	0.039 (0.003)	0.39 (0.04)	0.115 (0.008)	0.0630 (0.0002)	0.060 (0.009)	0.11 (0.01)	0.35 (0.02)	1.07 (0.03)	0.030 (0.002)	0.03 (0.00)	0.024 (0.004)	-	0.06 (0.00)	0.028 (0.003)	0.13 (0.01)	0.171 (0.007)
Sucrose	0.090 (0.003)	0.13 (0.02)	8.2 (0.9)	0.76 (0.06)	0.30 (0.03)	0.39 (0.03)	0.20 (0.03)	21.9 (3.3)	6.9 (0.6)	0.12 (0.01)	0.146 (0.001)	0.11 (0.01)	-	0.59 (0.04)	0.132 (0.007)	0.33 (0.02)	0.53 (0.07)
Maltose	20.00 (0.04)	22.3 (1.1)	2.4 (0.3)	5.378 (0.007)	35.8 (2.0)	0.5 (0.1)	0.49 (0.04)	0.33 (0.03)	3.5 (0.5)	21.6 (1.6)	14.2 (0.2)	12.3 (1.4)	-	0.45 (0.02)	12.9 (0.7)	0.21 (0.01)	0.23 (0.02)
Gentiobiose	0.11 (0.01)	0.18 (0.01)	0.100 (0.007)	0.50 (0.08)	0.50 (0.02)	0.22 (0.02)	0.307 (0.004)	0.162 (0.001)	2.9 (0.4)	0.271 (0.003)	0.13 (0.02)	0.13 (0.02)	-	0.19 (0.01)	0.16 (0.02)	0.48 (0.04)	0.50 (0.05)
Maltotriose	320.7 (39.6)	335.1 (51.0)	24.5 (0.8)	98.2 (7.7)	670.4 (10.0)	-	12.3 (1.5)	2.6 (0. 4)	40.0 (6.9)	463. 8 (21.2)	188.3 (3.7)	188. 8 (31.5)	-	4.9 (0.1)	167.0 (25.4)	1.1 (0.2)	-
Maltotetraose	211.8 (44.8)	340.5 (44.8)	-	-	400.1 (58.2)	-	-	-	-	319.7 (11.6)	163.7 (1.3)	155.1 (12.8)	-	-	133.6 (22.9)	-	-

^#^ Non-detected.

**Table 5:** SFS polar compounds quantified by LC-UV-MS (µg g^−1^ of SFS). Standard deviations in parentheses.

								SFS									
	1	2	3	4	5	6	7	8	9	10	11	12	13	14	15	16	17
Kaempferol-3-sophoroside-7-glucoside	53 (2)	66 (2)	18 (4)	NQ ^$^	280 (10)	-	130 (20)	95 (6)	1880 (80)	41.7 (0.2)	66.0 (3.4)	49 (1)	96.3 (0.7)	139.4 (0.5)	47 (2)	300 (3)	84 (4)
Picrocrocin	800 (20)	1010 (30)	- ^#^	3390 (80)	4300 (200)	-	2300 (300)	1928 (4)	36,000 (2000)	215 (2)	650 (30)	625 (6)	1010 (8)	2037 (4)	580 (20)	5060 (100)	1300 (40)
Kaempferol triglucoside	13.0 (0.3)	13.94 (1.06)	-	191.0 (10.0)	60.9 (3.0)	-	27.7 (4.0)	43.2 (0.8)	443.1 (21.5)	-	13.8 (0.39)	-	21.3 (1.2)	31.85 (0.02)	-	94.0 (0.6)	18.2 (1.4)
Kaempferol-7-sophoroside or Kaempferol-3,7-diglucoside	<*LOQ*	<*LOQ*	-	NQ	69 (2.7)	-	15.8 (3.1)	15.2 (0.4)	336.9 (12.3)	-	-	-	11.2 (0.1)	26.08 (1.09)	-	66.2 (2.7)	11.8 (0.9)
Kaempferol-3-sophoroside	99.2 (2.1)	127.4 (12.8)	-	528.3 (21.4)	471.1 (21.4)	-	265.1 (34.6)	404.7 (23.7)	4254.3 (162.5)	50.7 (0.3)	107.6 (7.3)	64.1 (1.1)	169.1 (1.1)	306.9 (3.8)	60 (2.6)	854.5 (16.3)	153.6 (11.8)
Kaempferol-3-glucoside	<*LOQ*	*<LOQ*	67.0 (4.1)	93.3 (5.6)	*<LOQ*	-	*<LOQ*	11.8 (0.9)	-	-	-	-	-	18.6 (2.5)	-	58.1 (4.5)	-
*trans*-5-tG	6.4 (0.5)	11.1 (1.4)	<*LOQ*	54.3 (2.2)	68.9 (2.3)	-	23.0 (3.5)	32.3 (0.8)	573.7 (28.5)	0.1 (0.1)	8.1 (0.7)	5.2 (0.1)	17.6 (0.1)	26.8 (0.2)	5.8 (0.4)	74.5 (0.8)	10.6 (1.1)
*trans*-5-nG	0.8 (0.4)	2.2 (0.5)	<*LOQ*	26.4 (0.5)	16.6 (0.7)	-	5.5 (0.9)	11.0 (0.6)	189.91 (17.6)	<*LOQ*	<*LOQ*	<*LOQ*	2.3 (0.1)	7.8 (0.1)	<*LOQ*	30.7 (0.7)	1.4 (0.7)
*trans*-4-GG	415.2 (21.4)	736.2 (36.4)	82.8 (1.4)	2896.9 (120.7)	38,423 (99)	-	1268.0 (137.2)	1733.6 (61.2)	32,703.3 (1267.3)	77.2 (2.7)	464.6 (31.6)	318.2 (1.2)	949.2 (3.5)	1393.6 (4.1)	370.3 (17.1)	3217.1 (33.2)	602.1 (26.9)
*trans*-4-tg	25.6 (1.7)	35.8 (3.5)	0.3 (0.2)	36.9 (1.7)	50.9 (10.1)	-	42.2 (6.8)	102.7 (0.4)	350.0 (13.8)	<*LOQ*	46.5 (1.9)	44.1 (1.1)	72.8 (0.8)	91.7 (0.7)	37.9 (1.3)	32.0 (0.3)	54.1 (0.1)
*trans*-4-ng	2.5 (0.6)	7.8 (1.5)	<*LOQ*	45.1 (3.8)	37.9 (1.2)	-	10.4 (2.3)	28.8 (0.9)	604.3 (16.6)	<*LOQ*	2.6 (0.4)	<*LOQ*	12.1 (0.3)	15.64 (0.04)	<*LOQ*	41.1 (0.2)	5.28 (0.02)
*trans*-3-Gg	159.1 (5.7)	283.0 (10.8)	25 (1)	1266.5 (54.1)	1516 (37)	-	473.1 (58.0)	625.1 (16.9)	12,074.9 (443.3)	25.45 (1.04)	252.1 (18.4)	163.7 (3.4)	591.5 (0.9)	533.6 (1.3)	205.9 (9.4)	1219.8 (14.5)	194.95 (8.05)
*trans*-2-gg	12.2 (0.8)	21.6 (1.4)	<*LOQ*	110.67 (1.7	142.8 (2.1)	-	39.8 (5.2)	48.0 (0.7)	663.2 (2.8)	0.40 (0.09)	15.0 (5.1)	9.0 (1.7)	34.1 (0.4)	42.8 (1.4)	10.9 (0.8)	101.5 (0.3)	17.71 (1.03)
*cis*-4-GG	60.1 (5.4)	99.5 (3.5)	10.1 (0.5)	294.3 (5.6)	486.4 (15.6)	-	235.7 (31.2)	308.7 (5.3)	3278.5 (100.4)	5.3 (0.3)	60.1 (6.6)	43.1 (0.9)	131.0 (1.7)	212.5 (6.3)	53.2 (2.3)	329.4 (1.1)	98.7 (3.9)
*cis*-4-ng	9.2 (1.3)	18.72 (2.04)	<*LOQ*	21.9 (0.1)	23.5 (1.2)	-	23.3 (3.4)	58.6 (0.5)	172.7 (7.6)	<*LOQ*	19.1 (1.3)	17.8 (0.2)	37.9 (0.4)	45.9 (0.7)	18.1 (0.7)	15.9 (0.6)	26.71 (0.02)
*trans*-2-G	74.8 (3.2)	118.6 (15.5)	12.5 (0.9)	444.1 (19.2)	380.2 (5.2)	-	259.1 (37.8)	435.1 (22.2)	4371.7 (156.5)	23.2 (0.6)	167.0 (8.8)	150.1 (3.1)	267.4 (2.0)	220.5 (1.3)	144.9 (4.9)	332.9 (1.0)	174.3 (3.6)
*cis*-3-Gg	18.6 (2.1)	33.9 (4.8)	<*LOQ*	110.8 (3.8)	176.4 (4.3)	-	87.9 (11.9)	117.2 (3.3)	1218.2 (40.9)	0.9 (0.1)	11.0 (1.8)	6.0 (0.1)	16.9 (2.0)	79.4 (2.7)	7.4 (0.2)	130.3 (0.7)	33.7 (1.5)
*cis*-2-gg	7.3 (0.6)	14.0 (1.7)	<*LOQ*	57.4 (2.0)	48.5 (1.8)	-	27.1 (4.6)	48.6 (2.1)	471.8 (11.9)	1.9 (0.3)	12.0 (2.2)	10.8 (0.3)	21.6 (1.8)	23.6 (1.2)	10.1 (0.2)	28.5 (1.2)	18.4 (0.4)
*trans*-1-g	5.3 (0.2)	9.86 (1.09)	<*LOQ*	23.3 (1.2)	21.3 (0.9)	-	26.9 (3.7)	40.9 (1.1)	160.3 (9.0)	<*LOQ*	25.5 (2.2)	22.3 (0.3)	44.0 (0.6)	25.4 (1.2)	21.5 (1.0)	15.6 (0.9)	21.28 (0.02)
*cis*-2-G	2.72 (0.07)	5.4 (0.6)	0.4 (0.4)	14.8 (0.1)	13.5 (0.6)	-	17.6 (2.5)	25.2 (0.1)	94.71 (1.9)	<*LOQ*	20.3 (1.0)	18.41 (0.09)	34.2 (0.4)	15.7 (0.4)	17.4 (0.7)	9.6 (1.3)	15.4 (0.1)
*cis*-1-g	8.3 (2.5)	<*LOQ*	<*LOQ*	<*LOQ*	15.3 (2.1)	-	7.8 (1.4)	6.2 (0.5)	<*LOQ*	<*LOQ*	18.46 (0.02)	22.5 (0.8)	8.3 (0.2)	2.8 (0.1)	7.87 (0.02)	<*LOQ*	15.7 (0.7)
*trans*-crocetin	0.9 (0.2)	2.6 (0.5)	<*LOQ*	11.7 (0.4)	13.7 (0.1)	-	10.0 (1.4)	14.8 (0.3)	76.5 (4.8)	1.41 (0)	38.5 (1.6)	37.6 (2.2)	43.2 (0.5)	3.0 (0.1)	33.6 (1.7)	<*LOQ*	17.5 (0.2)
*cis*-crocetin	<*LOQ*	<*LOQ*	<*LOQ*	<*LOQ*	<*LOQ*	-	<*LOQ*	0.48 (0.02)	<*LOQ*	<*LOQ*	6.2 (0.5)	5.1 (0.2)	9.1 (0.2)	<*LOQ*	4.0 (0.3)	<*LOQ*	<*LOQ*

^#^. Non-detected; ^$^ Not possible to quantitate due to coelution with other compounds.

## Data Availability

The data used to support the findings of this study can be made available by the corresponding author upon request.

## Supplementary Materials

The following supporting information can be downloaded at: https://www.mdpi.com/article/10.3390/foods12224071/s1, Figure S1. GC-MS chromatograms of saffron dried stigma (DS1) extract in dichloromethane (A) and water after derivatization (B). Peak identifications in Table S3. *: artefact; ** HTCC derivative; 13: unknown glycoside; 14: picrocrocin; Figure S2. Mass spectrum of trimethylsilyl oxime derivatized picrocrocin and proposed fragmentation pattern for characteristic fragment 237 *m/z*; Figure S3. (A) Principal component analysis (PCA) biplot of multi-analytical data for dried saffron stigmas (DS), commercial saffron extracts (SE), and food supplements (SFS) under study obtained by HPLC-DAD (Kaempferol glycosides, picrocrocin, crocins and safranal) and by GC-MS (volatiles, monosaccharides, *myo*-inositol, disaccharides and sugars from maltodextrins); (B) Projection of the variables on the factor plane (factor 1 vs. factor 2); Table S1. Characteristics and composition (data per formulation unit) of saffron food supplements (SFS) under study; Table S2. Concentration (mg g^−1^) of picrocrocin, crocin isomers, safranal and kaempferols of reference samples determined by LC-DAD. Standard deviations in parentheses (n = 2). *β*-D-glucopyranosyl (g), *β*-D-gentiobiosyl (G), *β*-D-neopolitanosyl (n), tri-*β*-D-glucopyranosyl (t). ID numbers correspond to peaks in Figure 1. Table S3. Concentrations of volatiles and low molecular weight carbohydrates (mg g^−1^) present in reference samples by GC-MS. Standard deviations in parentheses (n = 2).
