# Supplementary material for: A Combined Gas and Liquid Chromatographic Approach for Quality Evaluation of Saffron-Based Food Supplements
Source: Foods. 2023 Nov 9;12(22):4071. doi: 10.3390/foods12224071 (PMC10670060; doi:10.3390/foods12224071)

Table S1. Characteristics and composition (data per formulation unit) of saffron food supplements (SFS) under study.

SFS	Formulation	Saffron extract content (Bioactive concentration)	Other bioactive ingredients (Bioactive concentration)	Excipients
SFS1	Caplets	14 mg affron® (3.5 % Lepticrosalides®: 3.48 % crocins; 0.03% safranal)	None	Maltodextrins, talcum, food grade shellac, E-171, E-172, magnesium stearate
SFS2	Capsules	15 mg affron® (3.5 % Lepticrosalides®: 3.48 % crocins; 0.03% safranal)	Vitamin B6 (0.7 mg)	Maltodextrins, silicon dioxide
SFS3	Caplets	10 mg saffron dry extract (N.D.)	50 mg *Curcuma longa* dry extract (N.D.)<br>75 mg *Salix alba* bark dry extract (19 mg salicin)<br>162 mg Pinneapple stem dry extract (160 GDU Bromelain)<br>60 mg *Ribes nigrum* dry extract (N.D.)<br>200 mg *Boswellia serrata* rubber resin dry extract (N.D.)<br>Resveratrol (30 mg)<br>5 mg Black pepper dry extract<br>Q10 Coenzyme (10 mg)<br>Zinc oxide (12 mg Zn)<br>Copper sulphate (1000 mcg Cu)<br>Astaxanthin (1 mg)<br>Lutein (10 mg)<br>Zeaxanthin (1 mg)	Microcrystalline cellulose, dicalcium phosphate, sodium carboximethylcelulose, fatty acids mono and diglycerides, silicon dioxide, fatty acids magnesium salts, E1203, E1521, E553b, E101(i), E171, E172, E150d
SFS4	Capsules	60 mg saffron dry extract (2 % safranal)	200 mg *Garcinia cambogia* dry extract (60% hydroxycitric acid)<br>200 mg Green coffee dry extract (45% chlorogenic acid)	Maltodextrins, silicon dioxide, magnesium stearate
SFS5	Capsules	40 mg saffron 4:1 extract (2 % safranal)	Vitamin B6 (1.4 mg)	Maltodextrins, magnesium stearate, hypromellose, chlorophyll cupper complexes
SFS6	Capsules	180 mg saffron 4:1 extract (N.D.)	Vitamin C (26 mg)<br>Vitamin B6 (10 mg)	Magnesium stearate

			Vitamin B9 (200 µg)<br>Vitamin B12 (25 µg)<br>Vitamin B6 (30 µg)<br>Magnesium (101 mg)<br>L-Tyrosine (100 mg)<br>L-Tryptophan (100 mg)<br>Betain (26 mg)<br>Choline (10 mg)	
SFS7	Capsules	30 mg SAFFR'ACTIV ® saffron extract (2% safranal)	None	Microcrystalline cellulose, magnesium stearate pullulan
SFS8	Capsules	30 mg affron® saffron extract (3.5 % Lepticrosalides®: 3.48 % crocins; 0.03% safranal)	None	None
SFS9	Capsules	200 mg saffron extract (2 % safranal)	Melatonin (1.8 mg)<br>Vitamin B6 (1.1 mg)<br>L-Tyrosine (100 mg)<br>L-Tryptophan (N.D.)<br>Vitamin C (N.D.)<br>Betain (N.D.)	Maltodextrins, silicon dioxide, magnesium stearate
SFS10	Capsules	17 mg SAFFR'ACTIV ® saffron extract (2% crocin; 2 % safranal)	Vitamin B6 (1.4 mg)	Maltodextrins, silicon dioxide, hydroxypropylmethylcellulose
SFS11	Capsules	30 mg saffron extract (2 % safranal)	None	Microcrystalline cellulose, maltodextrins, silicon dioxide, magnesium stearate, hydroxypropylmethylcellulose
SFS12	Capsules	30 mg saffron extract (2 % safranal)	None	Microcrystalline cellulose, maltodextrins, silicon dioxide, magnesium stearate, hydroxypropylmethylcellulose
SFS13	Capsules	30 mg saffron extract (3 % crocins)	Trimagnesium citrate (65 mg)<br>Vitamin B5 (6 mg)<br>Vitamin B6 (1.4 mg)<br>Vitamin B9 (3 µg)	Gelatin, maltodextrins, rice extract, rice hulls, arabic gum, sunflower oil
SFS14	Caplets	28 mg affron® saffron extract (3 % crocins; 2 % safranal)	Vitamin B6 (1.4 mg)	Microcrystalline cellulose, dihydrated bicalcium phosphate, sodium carboximethylcelulose, silicon dioxide, magnesium stearate,

				hydroxypropylmethylcellulose, talcum, glycerine, titanium dioxide, red iron oxide and yellow iron oxide
SFS15	Capsules	15 mg saffron extract (2 % safranal)	None	Microcrystalline cellulose, maltodextrins, silicon dioxide, magnesium stearate, gelatin
SFS16	Caplets	30 mg saffron extract (4 % crocins; 2 % safranal)	None	Microcrystalline cellulose, silanized microcrystalline cellulose, silicon dioxide, polyvinylpyrrolidone
SFS17	Capsules	28 mg saffron extract (3.48 % crocins; 0.03% safranal)	*Lactobacillus acidophilus* (1000 millions) *Lactobacillus casei* (1000 millions) *Bifidobacterium bifidum* (1000 millions)	cellulose, hypromellose, silicon dioxide, magnesium stearate

*N.D.: non-declared safranal content.

Table S2. Concentration (mg g^{-1}) of picrocrocin, crocin isomers, safranal and kaempferols of reference samples determined by LC-DAD. Standard deviations in parentheses (n = 2). *β*-D-glucopyranosyl (g), *β*-D-gentiobiosyl (G), *β*-D-neopolitanosyl (n), tri-*β*-D-glucopyranosyl (t). ID numbers correspond to peaks in Figure 1.

	ID	SE1	SE2	SE 3	DS1	DS2	DS3	DS4	DS5
Kaempferol-3-sophoroside-7-glucoside	1	1.88 (0.05)	1.45 (0.02)	1.6 (0.05)	5,88 (0,03)	6,12 (0,03)	5.5 (0.1)	6.6 (0.2)	6.7 (0.2)
Picrocrocin	2	32.6 (0.7)	29.7 (0.5)	29.3 (0.9)	102.8 (1.4)	91.6 (0.1)	89.0 (2.9)	105.4 (2.9)	74.7 (1.0)
Kaempferol triglucoside	3	0.42 (0.02)	0.401 (0.006)	0.40 (0.01)	1.51 (0.04)	1.53 (0.01)	1.422 (0.007)	1.63 (0.03)	1.62 (0.02)
Kaempferol-7-sophoroside or Kaempferol-3.7-diglucoside	4	0.34 (0.02)	0.325 (0.005)	0.270 (0.006)	0.97 (0.06)	1.14 (0.02)	1.10 (0.01)	1.01 (0.04)	0.88 (0.07)
Kaempferol-3-sophoroside	5	4.1 (0.1)	4.03 (0.04)	3.9 (0.1)	13.0 (0.3)	13.8 (0.1)	13.0 (0.1)	14.1 (0.4)	13.5 (0.8)
Kaempferol-3-glucoside	6	0.171 (0.006)	0.14 (0.02)	0.13 (0.02)	0.85 (0.06)	0.730 (0.003)	0.65 (0.05)	0.69 (0.03)	0.98 (0.10)
trans-5-tG	7	0.41 (0.04)	0.36 (0.01)	0.37 (0.01)	1.64 (0.01)	1.81 (0.02)	1.50 (0.05)	1.72 (0.05)	1.42 (0.03)
trans-5-nG	8	0.12 (0.01)	0.118 (0.003)	0.108 (0.003)	0.48 (0.01)	0.446 (0.005)	0.50 (0.01)	0.36 (0.02)	0.44 (0.01)
trans-4-GG	9	20.5 (1.7)	19.9 (0.6)	20.8 (0.5)	82.9 (1.0)	89.0 (0.3)	86.8 (2.9)	95.6 (1.8)	76.7 (1.1)
trans-4-tg	10	1.63 (0.09)	0.91 (0.04)	1.02 (0.03)	0.80 (0.02)	0.75 (0.01)	0.83 (0.03)	0.97 (0.05)	0.77 (0.02)
trans-4-ng	11	0.39 (0.03)	0.33 (0.01)	0.31 (0.01)	1.71 (0.01)	1.88 (0.01)	1.7 (0.1)	1.9 (0.1)	1.33 (0.02)
trans-3-Gg	12	8.1 (0.8)	7.9 (0.2)	8.5 (0.3)	33.7 (0.5)	35.89 (0.2)	35.8 (1.3)	38.8 (1.0)	32.1 (0.6)
trans-2-gg	13	0.6 (0.1)	0.58 (0.01)	0.62 (0.01)	2.8 (0.1)	2.64 (0.04)	2.4 (0.1)	2.6 (0.1)	2.7 (0.1)
cis-4-GG	14	3.7 (0.3)	3.24 (0.09)	3.27 (0.09)	8.7 (0.2)	9.71 (0.02)	11.3 (0.3)	10. 2 (0.2)	8.485 (0.1)
cis-4-ng	15	0.50 (0.04)	0.51 (0.03)	0.55 (0.02)	0.41 (0.02)	0.373 (0.004)	0.39 (0.02)	0.42 (0.01)	0.40 (0.02)
trans-2-G	16	4.1 (0.3)	3.3 (0.2)	3.4 (0.1)	8.3 (0.2)	8.81 (0.01)	8.9 (0.3)	9.2 (0.2)	8.7 (0.1)
cis-3-Gg	17	1.4 (0.1)	1.22 (0.03)	1.25 (0.03)	3.5 (0.1)	3.77 (0.02)	4.6 (0.1)	4.0 (0.1)	3.5 (0.1)
cis-2-gg	18	0.49 (0.05)	0.39 (0.02)	0.44 (0.04)	1.03 (0.03)	1.14 (0.04)	1.16 (0.04)	1.13 (0.01)	1.301 (0.005)
trans-1-g	19	0.42 (0.04)	0.29 (0.01)	0.30 (0.01)	0.31 (0.01)	0.309 (0.001)	0.37 (0.01)	0.31 (0.02)	0.33 (0.01)
cis-2-G	20	0.28 (0.03)	0.18 (0.01)	0.18 (0.01)	0.180 (0.004)	0.184 (0.002)	0.23 (0.01)	0.18 (0.01)	0.19 (0.01)
cis-1-g	21	0.07 (0.01)	0.038 (0.003)	0.043 (0.002)	0.016 (0.005)	0.031 (0.001)	0.051 (0.004)	< LOQ	0.023 (0.003)
trans-crocetin	22	0.12 (0.02)	0.06 (0.02)	0.06 (0.01)	0.16 (0.02)	0.273 (0.002)	0.2 (0.01)	0.22 (0.01)	0.29 (0.02)
cis-crocetin	23	0.009 (0.004)	< *LOQ*	< *LOQ*	-[#]	-	-	-	-

Safranal	*	0.50 (0.03)	0.35 (0.01)	0.27 (0.01)	3.08 (0.05)	2.52 (0.01)	2.6 (0.1)	2.69 (0.05)	3.30 (0.04)

non detected.

Table S3. Concentrations of volatiles and low molecular weight carbohydrates (mg g^{-1}) present in reference samples by GC-MS. Standard deviations in parentheses (n = 2).

	ID	SE1	SE2	SE 3	DS1	DS2	DS3	DS4	DS5
Dihydro-4-hydroxy-2(3H)-furanone	1	0.008 (0.001)	0.002 (0.003)	0.005 (0.001)	0.07 (0.02)	0.11 (0.01)	0.14 (0.03)	0.12 (0.03)	0.09 (0.01)
Isophorone	2	0.013 (0.002)	0.008 (0.004)	0.008 (0.001)	0.23 (0.004)	0.20 (0.01)	0.24 (0.04)	0.24 (0.01)	0.23 (0.01)
Ketoisophorone	3	0.006 (0.001)	0.004 (0.001)	0.002 (0.003)	0.17 (0.01)	0.11 (0.01)	0.121 (0.005)	0.131 (0.004)	0.117 (0.005)
Dihydrooxophorone	4	0.01 (0.0001)	0.006 (0.003)	0.007 (0.001)	0.20 (0.01)	0.199 (0.005)	0.2 (0.005)	0.208 (0.002)	0.18 (0.01)
4-Hydroxy-isophorone	6	0.014 (0.001)	0.009 (0.004)	0.012 (0.001)	0.17 (0.01)	0.14 (0.01)	0.18 (0.03)	0.202 (0.02)	0.16 (0.01)
4-Hydroxy-2,6,6-trimethyl-3-oxocyclohexa-1,4-diene-1-carboxaldehyde	7	0.007 (0.001)	0.01 (0.01)	0.005 (0.003)	0.26 (0.05)	0.17 (0.01)	0.2 (0.1)	0.25 (0.01)	0.19 (0.01)
HTCC	8	0.034 (0.001)	0.022 (0.01)	0.027 (0.001)	0.46 (0.03)	0.31 (0.01)	0.5 (0.1)	0.59 (0.01)	0.40 (0.02)
Fructose*	9	2.1 (0.1)	1.4 (0.3)	1.2 (0.1)	13.0 (0.1)	12.05 (0.05)	12.4 (0.2)	12.1 (0.8)	12.0 (0.1)
Glucose*	10	11.8 (1.1)	7.0 (0.3)	5.5 (0.1)	29.0 (1.0)	50.0 (0.3)	41.4 (1.0)	44.3 (2.9)	30.8 (1.0)
myo-Inositol*	11	1.01 (0.01)	0.75 (0.02)	0.4 (0.3)	2.78 (0.07)	2.6 (0.1)	2.45 (0.1)	2.6 (0.2)	2.85 (0.03)
Sucrose*	12	9.2 (0.1)	7.3 (0.6)	7.4 (0.1)	4.1 (0.1)	2.1 (0.4)	2.0 (1.9)	3.23 (0.01)	4.7 (0.6)
Maltose*	15	7.0 (0.7)	3.5 (0.1)	3.7 (0.2)	ND	ND	ND	ND	ND
Gentiobiose*	16	5.6 (0.1)	1.5 (0.1)	1.27 (0.03)	7.78 (0.1)	3.4 (0.9)	4.0 (0.3)	3.75 (0.04)	8.2 (1.2)
Maltotriose*	17	68.5 (12.5)	38.0 (7.8)	28.8 (2.7)	-[#]	-	-	-	-

* derivatized compounds; [#]: non detected

Figure S1. GC-MS chromatograms of saffron dried stigma (DS1) extract in dichloromethane (A) and water after derivatization (B). Peak identifications in Table S3. *: artefact; ** HTCC derivative; 13: unknown glycoside; 14: picrocrocin.

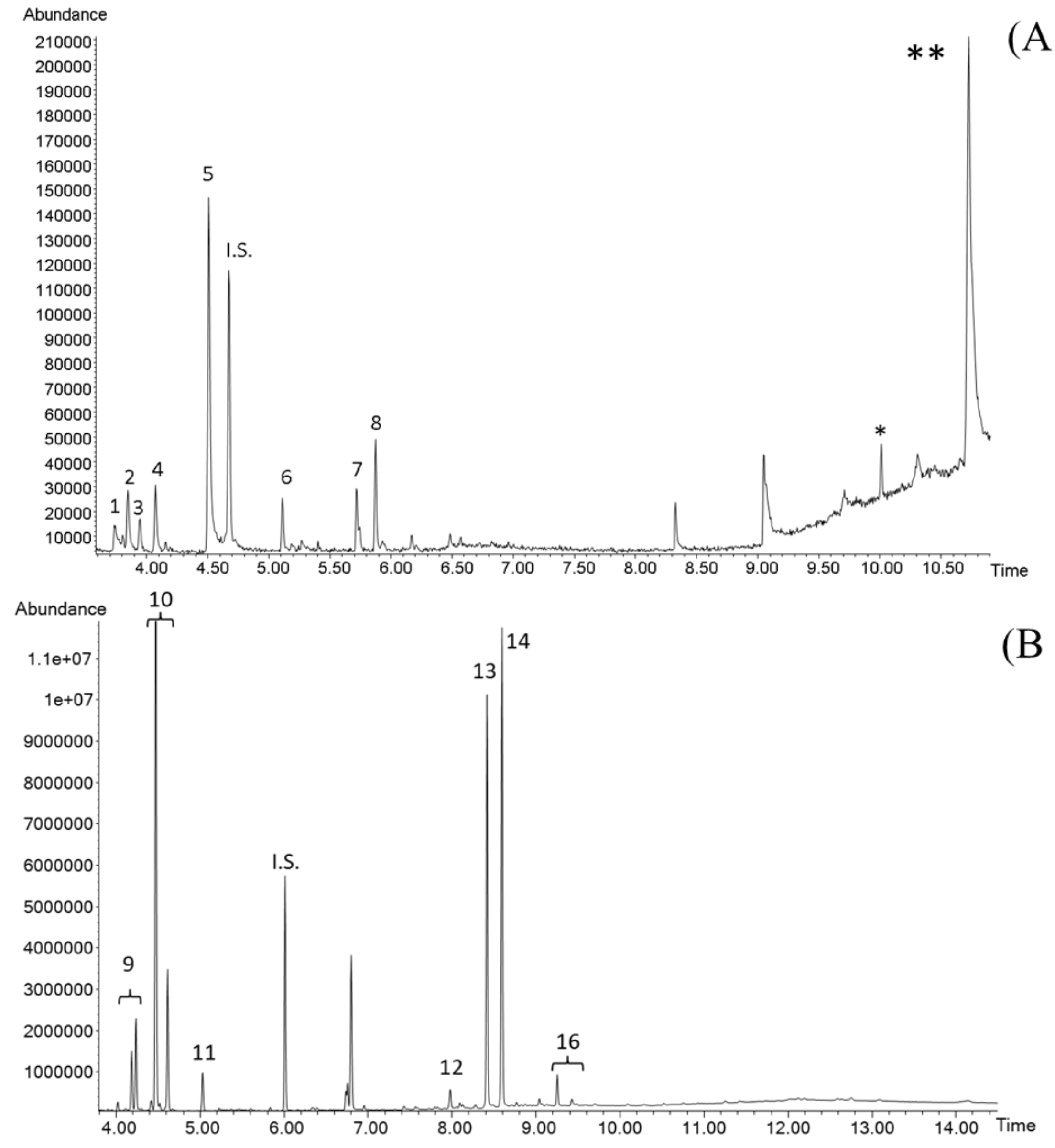

Figure S2. Mass spectrum of trimethylsilyl oxime derivatized picrocrocin and proposed fragmentation pattern for characteristic fragment 237 *m/z*.

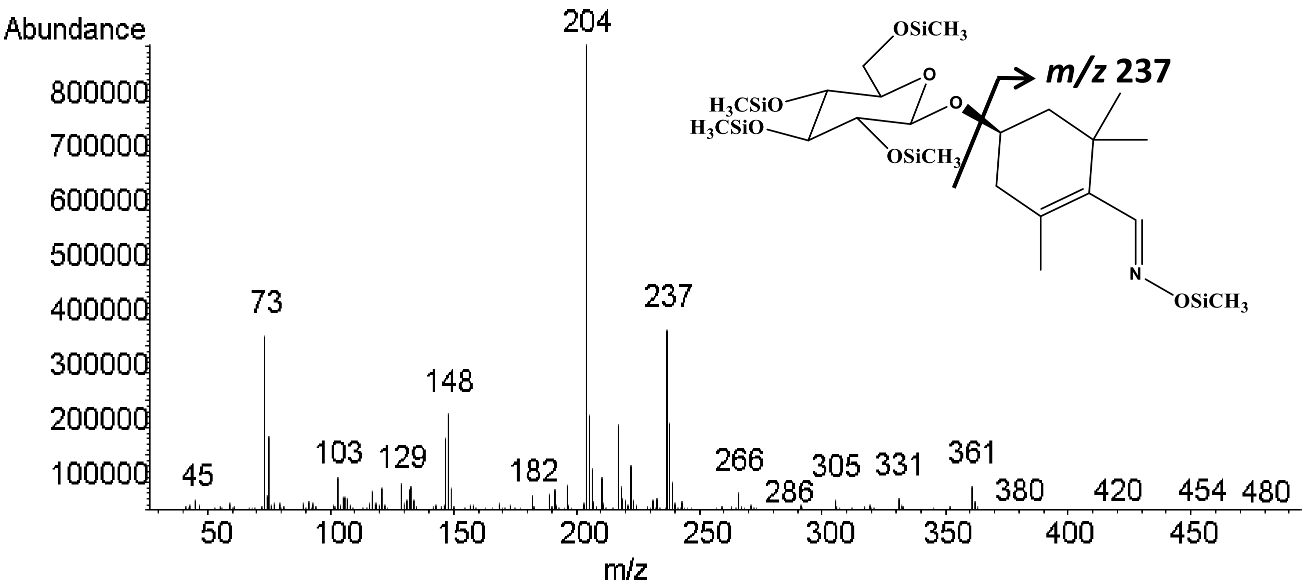

Figure S3. (A) Principal component analysis (PCA) biplot of multi-analytical data for dried saffron stigmas (DS), commercial saffron extracts (SE), and food supplements (SFS) under study obtained by HPLC-DAD (Kaempferol glycosides, picrocrocin, crocins and safranal) and by GC-MS (volatiles, monosaccharides, *myo*-inositol, disaccharides and sugars from maltodextrins); (B) Projection of the variables on the factor plane (factor 1 *vs*. factor 2).

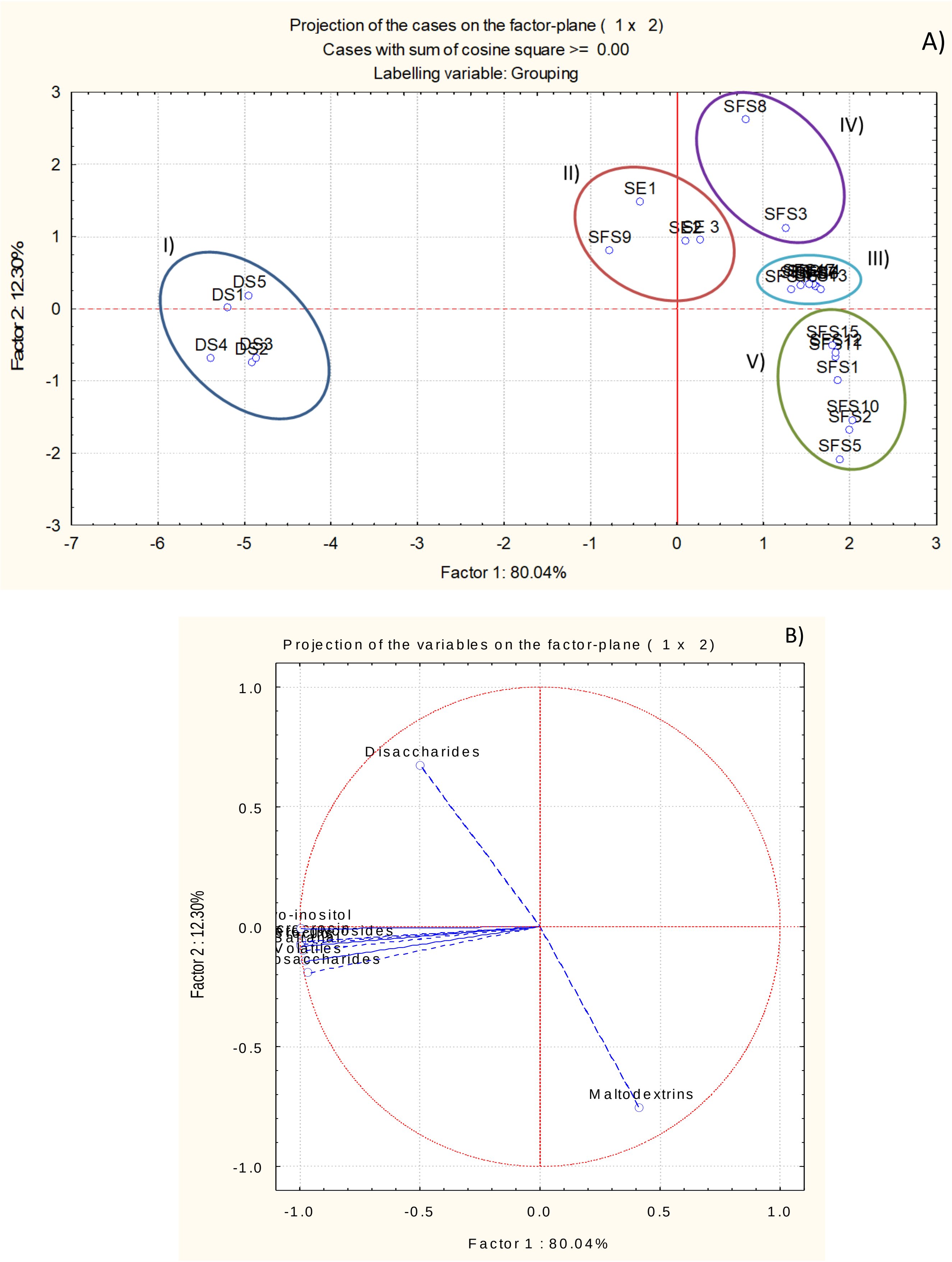

Supplement: Supplementary file 1 [file foods-12-04071-s001.zip › foods-2665341-supplementary.pdf]
